# Evaluation of Convolutional Neural Network-Based Posture Identification Model of Older Adults: From Silhouette of Sagittal Photographs

**DOI:** 10.3390/geriatrics10020049

**Published:** 2025-03-19

**Authors:** Naoki Sugiyama, Yoshihiro Kai, Hitoshi Koda, Toru Morihara, Noriyuki Kida

**Affiliations:** 1 Department of Advanced Fibro-Science, Kyoto Institute of Technology, Hashikami-cho, Matsugasaki, Sakyo-ku, Kyoto 606-8585, Japan; nsugiyama.u@gmail.com; 2 Department of Physical Therapy, Faculty of Health Sciences, Kyoto Tachibana University, 34 Yamada-cho, Oyake, Yamashina-ku, Kyoto 607-8175, Japan; kai-y@tachibana-u.ac.jp; 3 Department of Rehabilitation Sciences, Faculty of Allied Health Sciences, Kansai University of Welfare Sciences, Asahigaoka 3-11-1, Kashiwara-shi 582-0026, Japan; h-koda@tamateyama.ac.jp; 4 Marutamachi Rehabilitation Clinic, Nishinokyo Kurumazakacho Nakagyo-ku, Kyoto 604-8405, Japan; toru4271@koto.kpu-m.ac.jp; 5 Faculty of Arts and Sciences, Kyoto Institute of Technology, Hashikami-cho, Matsugasaki, Sakyo-ku, Kyoto 606-8585, Japan

**Keywords:** healthy life expectancy, posture assessment, convolutional neural networks

## Abstract

**Background/Objectives**: Posture is a significant indicator of health status in older adults. This study aimed to develop an automatic posture assessment tool based on sagittal photographs by validating recognition models using convolutional neural networks. **Methods**: A total of 9140 images were collected with data augmentation, and each image was labeled as either Ideal or Non-Ideal posture by physical therapists. The hidden and output layers of the models remained unchanged, while the loss function and optimizer were varied to construct four different model configurations: mean squared error and Adam (MSE & Adam), mean squared error and stochastic gradient descent (MSE & SGD), binary cross-entropy and Adam (BCE & Adam), and binary cross-entropy and stochastic gradient descent (BCE & SGD). **Results**: All four models demonstrated an improved accuracy in both the training and validation phases. However, the two BCE models exhibited divergence in validation loss, suggesting overfitting. Conversely, the two MSE models showed stability during learning. Therefore, we focused on the MSE models and evaluated their reliability using sensitivity, specificity, and Prevalence-Adjusted Bias-Adjusted Kappa (PABAK) based on the model’s output and correct label. Sensitivity and specificity were 85% and 84% for MSE & Adam and 67% and 77% for MSE & SGD, respectively. Moreover, PABAK values for agreement with the correct label were 0.69 and 0.43 for MSE & Adam and MSE & SGD, respectively. **Conclusions**: Our findings indicate that the MSE & Adam model, in particular, can serve as a useful tool for screening inspections.

## 1. Introduction

Japan has the highest aging rate worldwide, with 29% of its population aged 65 years or older [1,2]. Consequently, both lifespan and healthy life expectancy (HALE) are of paramount importance in Japan [3]. HALE refers to “the period during which a person can live without being limited in daily life by health problems” [4]. Extending HALE not only increases lifespan but also improves the quality of life (QOL) of older adults [5]. Among the several measures that can enhance QOL, maintaining and improving muscle strength is strongly recommended by the Ministry of Health, Labor and Welfare to prevent bedridden conditions [6,7,8]. Since muscle strength can be assessed through posture, assessing posture is a useful indicator of the physical health of older adults [9,10,11]. Additionally, posture is closely associated with mental health [12], and can reflect a wide range of health conditions.

Traditionally, posture assessment is conducted in a face-to-face environment by physical therapists (PTs) who rely on palpation and visual inspection. The criteria for posture assessment vary depending on the definition of “Ideal posture”. One widely recognized method is the Kendall classification [13], which categorizes posture into four types: Ideal posture and three forms of poor posture (kyphosis–lordosis (KL), sway back (SB), and flat back (FB)). These postures are characterized as follows: Ideal posture minimizes strain on muscles and bones; KL is marked by hyperkyphosis of the thoracic spine, hyperlordosis of the lumbar spine, and anterior tilt of the pelvis; SB involves hyperkyphosis of the thoracic spine, flattening of the lumbar spine, and posterior tilt of the pelvis; and FB is defined by flexion of the upper thoracic spine, flattening of the lower thoracic spine, flattening of the lumbar spine, and posterior tilt of the pelvis. PTs’ assessments are accurate, and the demand for testing is expected to increase as the number of older adults rises. However, face-to-face inspections are expensive and time-consuming, limiting the number of people who can be assessed. Consequently, opportunities for early detection are missed, and older adults may not take appropriate preventive measures. Although it has been reported that kyphosis index and occiput-to-wall distance can be used as simple methods for posture assessment at home [14], it is difficult for older adults to perform these tasks appropriately themselves. Therefore, developing an automatic posture assessment tool is necessary. We believe that one potential method for this development is using deep neural networks (DNNs).

Currently, DNNs are being applied across various fields. In particular, convolutional neural networks (CNNs) for image recognition have demonstrated accuracy comparable to or even surpassing that of human performance [15]. The medical field is no exception, with CNNs being used for analyzing a wide range of imaging data, such as magnetic resonance imaging (MRI) [16], computed tomography (CT) [17], X-ray [18], and mammography [19].

Several studies have been made on combining posture with DNNs, utilizing sensor-based techniques such as infrared maps and pressure maps, as well as image-based techniques like X-rays and video recordings [20,21]. Nowadays, most of the evaluations are conducted on time series using human pose estimation, such as OpenPose [22,23,24,25]. In a study using OpenPose, Barberi et al. developed a correctness identification model for isometric squat [26], and also proposed a method to enable the detection of posture changes by 3D reconstruction from human pose estimation in motorbike riding forms [27]. However, in order to develop practical and simple tools, it is important to consider the ease of posing and the usability of data acquisition methods when selecting input information. Therefore, we focused on posture assessment using static data from single images rather than time series data from videos. Regarding only visual inspection, according to Inae C. Gadott et al., standing posture can be assessed with a certain degree of reliability from photographs [28]. In addition, a previous study found that PTs can reliably identify Ideal or Non-Ideal posture through visual inspection [29]. This finding suggests that standing posture can be potentially assessed automatically using images. In fact, in the case of sitting posture, the effectiveness of photograph-based identification models for automatic assessment has been reported [30,31]. Therefore, this study aimed to develop an assessment tool to classify posture from images of older adults. In this article, we evaluate the performance of four general CNN models, based on convolutional layers, pooling layers, and fully connected multilayer perceptrons (MLPs), using different loss functions and optimizers. Demonstrating the efficacy of these models can contribute to the field of preventive medicine and personalized healthcare from the perspective of screening inspection, such as early detection of risks through daily posture checks. Furthermore, since static data collection via photography is easily accessible, this approach serves as a simple yet effective assessment tool.

## 2. Methods

### 2.1. Raw Images and Correct Labels in Supervised Data

The supervised data for the CNNs were gathered from a fitness event for older adults held in September 2018 and 2019. During this event, standing posture photography of older adults aged 40 to 93 years (75 ± 6.3) was collected. For the photography, participants were instructed to stand on a marked spot on the floor with their feet shoulder-width apart and their arms resting vertically along their bodies. The right sagittal plane of each participant was captured using a Kinectv2 color camera, which was positioned 3 m away to capture the entire body. There were no specific clothing requirements. One or two photographs were taken per person. As a result, a total of 656 photographs (119 men and 537 women) were gathered, including 84 duplicates. Since these duplicate images exhibit subtle variations in pose and angle, raw images were included to enhance the variety of the dataset. Participation in the event was voluntary.

Three PTs, each with over 10 years of clinical experience, conducted the posture assessments. The PTs aligned their posture assessment criteria prior to measurement to ensure consistency and high reliability in the assessment. Older adults were diagnosed according to the Kendall classification, which includes four categories: Ideal, KL, SB, and FB. These photographs and posture assessments were used as raw images and correct labels for the supervised data. The study was approved by the Ethics Committee of the Kyoto Institute of Technology (protocol no. 2018-19).

### 2.2. Pre-Processing of Raw Images

**Figure 1:**
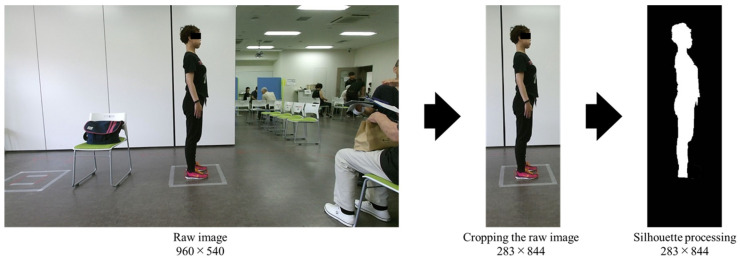
The workflow from the raw images captured during the event to the silhouette processing stage.

Pre-processing is a crucial technique for facilitating smooth learning in the construction of CNNs [32]. The raw images captured at a fitness event were RGB images containing substantial information, some of which could introduce noise into posture identification. Such noise is generally undesirable as it can lead to overfitting [33,34]. Therefore, the first pre-processing step was to crop the images to 283 × 844 pixels, focusing on the sagittal plane of the older adults. The second step involved applying silhouette processing to the cropped images. In a previous study, silhouette images were used as supervised data in DNNs [35], indicating the effectiveness of silhouette images for CNNs. Silhouette processing was performed using Photoshop’s subjective contour extraction function or codes for semantic segmentation by Fisher Yu and Vladlen Koltun [36]. Since the manual operation captured the contours of the neck and waist more accurately than the automated operation, silhouette processing was applied using Photoshop. The resulting silhouette images depicted older adults painted in white, with the background in black, and excluded the toes to remove depth information. Figure 1 shows the workflow from the raw images captured during the event to the silhouette processing stage.

Subsequently, any silhouette images with unclear body contours caused by clothing or accessories were removed, leaving 457 images in which the curvature of the back and the position of the waist were clearly indicated (Ideal: 207; KL; 43; SB: 152; FB: 55).

Subsequently, the 457 images underwent resizing and data augmentation, which are typical pre-processing techniques [37]. Resizing helps reduce computational complexity [38], while data augmentation enhances models’ generalization performance. In this experiment, the silhouette images were resized from 283 × 844 to 71 × 211, reducing them to a quarter of their original size. The resized images were then subjected to data augmentation in the following order: In the first processing step, enlargement and reduction were applied twice for each resized image. In the second process, translating up, down, right, and left was applied twice for resized, enlarged, and reduced images. Thus, one resized image was enhanced to 20 images through data augmentation. The parameters for these processes were selected randomly, ensuring that the posture in the image remained intact. A total of 9140 images were obtained (Ideal: 4140; KL: 860; SB: 3040; FB: 1100). The images obtained were then randomly divided using the holdout method into 60% for the training set, 20% for validation, and 20% for testing, with correct labels classified into two categories: Ideal and Non-Ideal (KL, SB, FB). In regard to the use of two categories, there are limitations to identifying Non-Ideal postures based on appearance, so it is likely that multi-classification models would have difficulty in distinguishing these postures. In addition, the correct labels were divided into two categories of Ideal and Non-Ideal based on previous studies [28,29].

### 2.3. Construction Model

**Figure 2:**
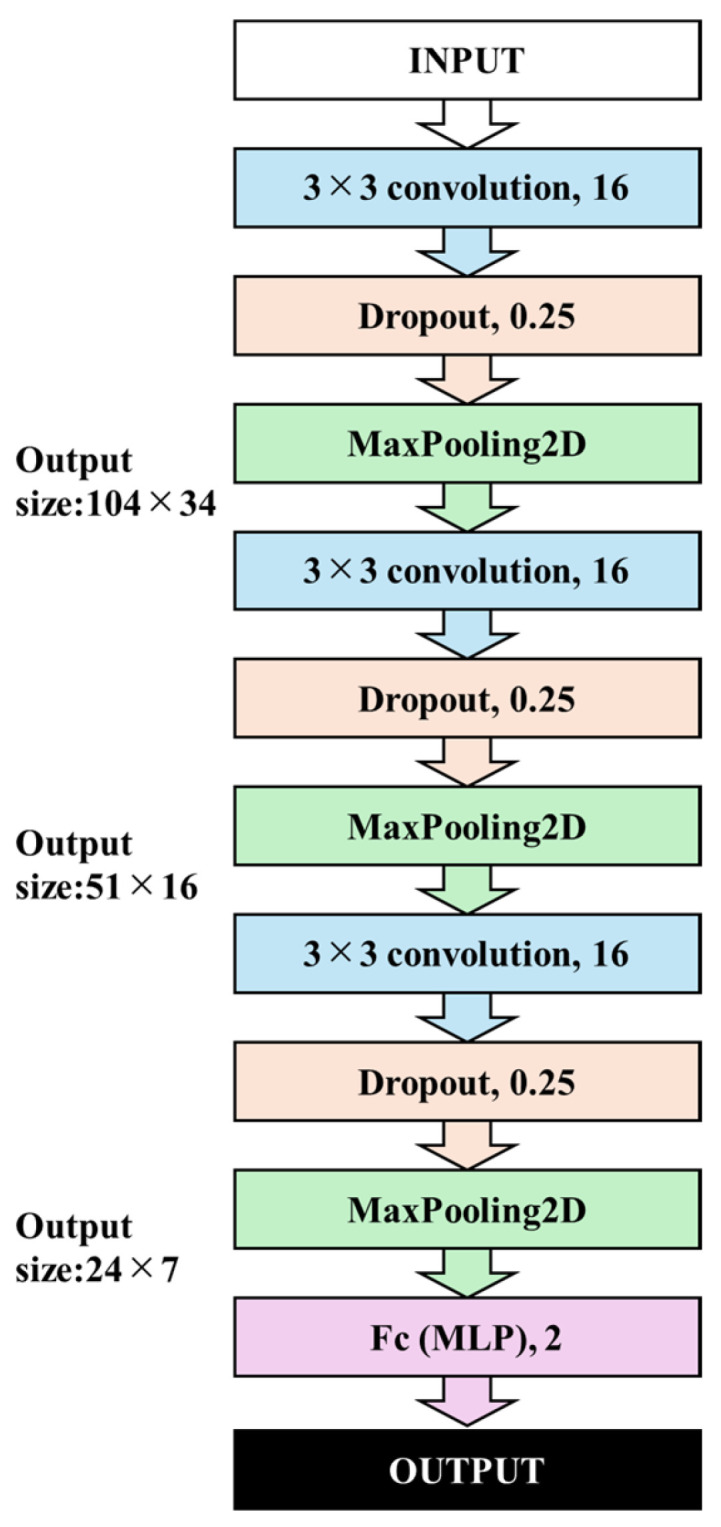
The CNN architecture.

The recognition models were constructed using CNNs, which are suitable for computer vision tasks [39]. CNNs consist of three primary layers: multiple convolution layers, pooling layers, and fully connected multilayer perceptrons (MLPs) [40]. In the construction model, three primary layers were utilized. Figure 2 illustrates the CNN architecture.

The input layer was fed with randomly selected images from the training set. The hidden layer consists of a combination of convolution, activation function, and pooling, repeated three times. Based on exploratory experiments, a transfer learning model based on VGG16 [41] and a general model with one to three primary layers were constructed and their architectures were compared. It was determined that using three layers provided more stability than using transfer learning and one or two layers; thus, the number of primary layers was set to three. The output layer comprises two layers corresponding to the correct labels. The activation function for each layer was a Rectified Linear Unit (ReLU), commonly used in DNNs [42,43]. Additionally, dropout and L2 normalization were applied to prevent overfitting [44,45,46,47], with the dropout set to 0.25.

In this experiment, the hidden and output layers of the models remained unchanged, while the loss function and optimizer were varied to evaluate four different model configurations. Regarding the loss function, mean squared error (MSE), binary cross-entropy (BCE), and categorical cross-entropy (CE) are commonly used [46,47]. Therefore, MSE and BCE were chosen for the models’ construction in this study. Regarding the optimizer, Adam is widely used in DNNs and CNNs, while stochastic gradient descent (SGD) has the advantage of preventing the model from falling into local minimum [48]. Therefore, both Adam and SGD were used in this study. Consequently, the four combinations evaluated were MSE & Adam, MSE & SGD, BCE & Adam, and BCE & SGD. The number of training epochs was set to 200, with a batch size of 32. The development environment used for the model was the language Python (ver. 3.6.10) and the deep learning library Keras (ver. 2.3.1, backend TensorFlow) [49,50]. Table 1 provides details of the main library versions used for the model development.

### 2.4. Evaluation Models

To evaluate the performance of the constructed models, we compared the correct and output labels in the test set of 1828 images. In addition, a cross-tabulation matrix was created between the correct and output labels. Sensitivity and specificity were calculated to determine the models’ ability to accurately identify true-negative and -positive results from the cross-tabulation matrix, providing insight into the reliability of both positive and negative predictions. Moreover, the consistency of posture assessment was evaluated using Prevalence-Adjusted Bias-Adjusted Kappa (PABAK) [51,52]. The PABAK value can be defined as follows: <0.00 = no agreement; 0.00–0.20 = slight agreement; 0.21–0.40 = fair agreement; 0.41–0.60 = moderate agreement; 0.61–0.80 = substantial agreement; and 0.81–1.00 = almost perfect agreement [53].

## 3. Results

### 3.1. Accuracy and Loss in Models Construction

**Figure 3:**
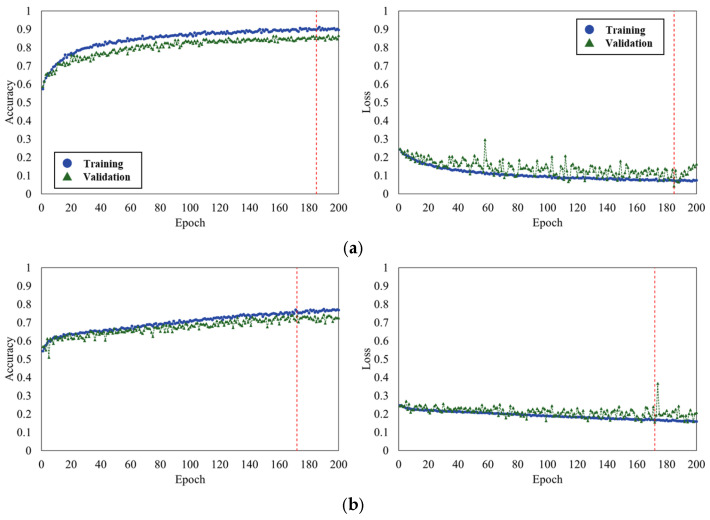

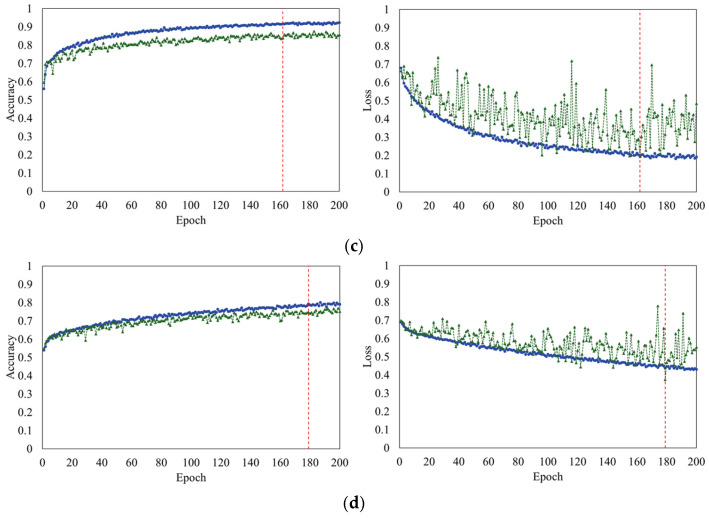
The learning curves for the four models: (**a**) MSE & Adam; (**b**) MSE & SGD; (**c**) BCE & Adam; (**d**) BCE & SGD.

The models’ training performance was evaluated using learning curves, which track training and validation accuracy, and loss over each epoch. Figure 3 presents the learning curves for the four models.

Accuracy is the percentage of correct answers in the training set, while validation accuracy is the percentage of correct answers in the validation set. Similarly, loss represents the error between predictions and correct answers in the training set, and validation loss represents the errors in the validation set.

According to the criterion of the lowest value of validation loss, the number of epochs was found to be 185 for MSE & Adam, 172 for MSE & SGD, 162 for BCE & Adam, and 179 for BCE & SGD. In the case of training, the accuracy and loss values were 0.90 and 0.07 for MSE & Adam, 0.76 and 0.16 for MSE & SGD, 0.92 and 0.20 for BCE & Adam, and 0.79 and 0.44 for BCE & SGD. For validation, the accuracy and loss values were 0.86 and 0.04 for MSE & Adam, 0.74 and 0.16 for MSE & SGD, 0.84 and 0.19 for BCE & Adam, and 0.75 and 0.37 for BCE & SGD. Notably, the models trained with the Adam optimizer demonstrated highest accuracy in both training and validation. In contrast, MSE & Adam also showed the lowest loss values during both the training and validation phases.

The results indicate that accuracy improved across all models. However, the validation loss showed a tendency to diverge in the learning curves of the BCE models. Since the divergence of the validation loss suggests a possibility of overfitting, we focused on two models: MSE & Adam and MSE & SGD.

Additionally, the MSE models were further evaluated for precision and recall. Precision and recall are defined by the following equations:

Precision = (True Positive)/(True Positive + False Positive)

Recall = (True Positive)/(True Positive + False Negative)

The two evaluation values were higher for MSE & Adam (precision: 0.90; recall: 0.90) than for MSE & SGD (precision: 0.76; recall: 0.76).

### 3.2. Agreement of Output and Correct Label Using Test Set

To evaluate the generalization performance of the constructed models, we compared the output labels of the MSE models with the correct labels in 1828 images in the test set. Table 2 shows the cross-tabulation matrix of the correct and output labels across different datasets. Moreover, Table 3 illustrates the performance metrics (accuracy, sensitivity, and specificity) of the two models.

Both models achieved the highest accuracy, sensitivity, and specificity in the training set. Regarding the validation and test sets, sensitivity was slightly higher in the test set, whereas accuracy and specificity were higher in the validation set. Since recall and sensitivity measure the same performance aspect, when comparing the two values in the test set, they were found to be slightly lower for MSE & Adam, and they were even lower for MSE & SGD. Additionally, based on Landis’ criteria, MSE & Adam showed substantial agreement (*p* < 0.001; kappa = 0.69; PABAK = 0.69), while MSE & SGD showed moderate agreement (*p* < 0.001; kappa = 0.43; PABAK = 0.43).

## 4. Discussion

### 4.1. Performance in the Four Models

To investigate the possibility of automatic posture assessment in older adults, we constructed four models using CNNs. The learning curves showed the improved accuracy of these models, indicating that learning progressed well. In previous studies, an automatic identification model for sitting posture achieved an accuracy of over 80% [30,31]. It is considered that a similar level of identification was achieved for standing posture in this study. However, it is necessary to consider not only accuracy but also generalization performance when constructing models [54]. One indicator of generalization performance is validation loss [55]. In this study, the validation loss of the models using MSE had more stable values than those using BCE. BCE is commonly used in classification problems [46,47], whereas MSE has been reported to perform comparably to BCE in the field of computer vision [56]. Therefore, although BCE is typically used in classification models, the most appropriate loss function should be selected based on the nature of supervised data. In the case of posture assessment, MSE demonstrated better generalization performance than BCE.

Additionally, precision and recall were calculated to evaluate the performance of the MSE-based models. Precision and recall for MSE & Adam were higher than for MSE & SGD. This suggests that MSE & Adam can accurately distinguish between Ideal and Non-Ideal postures in the images in the training and validation sets.

Consequently, although the models using BCE as the loss function show good learning progress, they have a problem with poor generalization performance. Therefore, the BCE models find it difficult to recognize unknown data. In contrast, the models using MSE indicate sufficient learning progress and are reliable in correctly identifying posture.

### 4.2. Performance of the Two MSE Models Using Test Set

In this study, the output labels for 1828 test set were compared with the correct labels to evaluate the performance of the MSE models. The differences were analyzed using a cross-tabulation matrix. The sensitivity and specificity of MSE & Adam were 85% and 84%, respectively, while those of MSE & SGD were 67% and 77%. Although the performance on the test set did not reach that of the training set, the test set demonstrated slightly higher sensitivity than the validation set, with accuracy and specificity showing comparable values. When comparing recall and sensitivity, they were slightly lower for MSE & Adam, and even lower for MSE & SGD. Although these models were slightly less accurate on the test set than on the training and validation sets, in particular, the MSE & Adam configuration is considered to function similarly when handling unknown data. Additionally, in light of the evaluation by PTs in a previous study (sensitivity: 86%; specificity: 57%) [29], MSE & Adam and MSE & SGD had lower sensitivity but higher specificity than the PTs’ evaluation. Moreover, the PABAK values were 0.69 and 0.43 for MSE & Adam and MSE & SGD, respectively. As with sensitivity and specificity, when compared with the PTs’ evaluation (PABAK: 0.57), MSE & Adam was higher, and MSE & SGD was lower. This indicates that MSE & Adam is sufficiently reliable for recognizing even unknown posture images.

In the medical field, many studies have reported the construction of models for CT and MRI images [16,17]. These images characteristically contain a lot of information. On the other hand, silhouette images have less information and more noise. However, these results suggest that MSE & Adam, when using silhouette images, seemed to achieve recognition accuracy comparable to that of PTs. Therefore, MSE & Adam appears to be useful for automatic posture assessment during screening inspections.

### 4.3. Limitation

It is necessary to discuss the limitations of the silhouette images used in this study. The images used as supervised data were silhouette images with clear body contours. This means that the supervised data did not include images with unclear contours. Therefore, it is possible that the constructed models may struggle to assess posture depending on the clothing of the older adults. Moreover, contours are also affected by sex and hairstyle. To address the issue of images with unclear contours, increasing the number of images or devising a fill method will be necessary. However, we consider that the MSE & Adam model can serve as a standard model construction.

Furthermore, the dataset must be discussed. In this study, data augmentation was performed on 457 images, including duplicate images. However, since the older adult in each raw image is the same, the model may be biased toward certain features. In addition, since these images were randomly divided, there is a higher potential for bias. Because of this, further diversification of data is needed. Nevertheless, the proposed model has shown a certain degree of effectiveness with the test set divided using the holdout method and can be used for fine-tuning. Therefore, by acquiring new data and expanding the dataset, we consider that the bias problem can be reduced and the accuracy of the model can be improved. However, for future studies, k-fold cross-validation should be used instead of the holdout method to ensure robustness.

Furthermore, the limitations of the model structure must be discussed. In this study, three primary layers were found to be more stable than one or two layers, so the number of layers was set to three. However, there are countless choices for the number of layers and hyperparameters, making it difficult to find the optimal values [55]. Although the constructed models are not optimal, they demonstrate considerable validity in generalization performance and accuracy. Additionally, these models may improve through fine-tuning, adjusting the learning rate, and tweaking the batch size.

Furthermore, the limitations of recognition must be addressed. Posture is assessed based on the kyphosis of bones and inner muscles [57,58]. Since the constructed models rely on images for identification, they are limited to postures that can be evaluated based solely on appearance. This means that the assessment may not be appropriate for postures that are difficult to identify visually. Furthermore, predicting the degree of posture change is challenging. However, the constructed models can assess large categories such as Ideal or Non-Ideal. We believe that there is potential to identify and predict complex postures by adding information into the images or by further subdividing the label definitions. In particular, by incorporating human pose estimation using OpenPose [24,25], it is possible to include not only the body shape but also the body structure in the data. Moreover, the 2D coordinates obtained from OpenPose can be extended to 3D reconstruction [27,59]. Adding such silhouette-based features may help to reduce classification errors.

Furthermore, the limitations regarding the feasibility of these models in a clinical environment must be discussed. The silhouette images were manually created using Photoshop’s subjective contour extraction function. Although taking photographs in a clinical environment is relatively easy, creating silhouette images poses significant challenges. However, using semantic segmentation in deep learning makes it possible to automatically segment human and non-human regions. We performed silhouette processing both manually and automatically in the pre-processing phase. However, the boundary between humans and the background could not be precisely delineated. At this stage, achieving this automatically remains challenging. Nonetheless, creating silhouette images seems feasible in the future. Once the silhouette images are obtained, the constructed models can be used for automatic posture assessment.

Finally, while the constructed models are not optimal, the MSE & Adam model is particularly useful for automatic posture assessment. Additionally, it can be used for transfer learning and fine-tuning.

## 5. Conclusions

This study aimed to develop an automatic posture assessment tool and investigate the performance of recognition models using CNNs. Four models were constructed, MSE & Adam, MSE & SGD, BCE & Adam, and BCE & SGD, each utilizing different loss functions and optimizers. The following results were obtained:

All four models demonstrated an improvement in accuracy for both the training and validation datasets. However, the two BCE models tended to diverge in terms of validation loss, suggesting potential overfitting. Conversely, the two MSE models showed stability during the learning process. In particular, MSE & Adam achieved high values for precision and recall. This suggests that posture recognition is possible in both the training and validation sets.

Additionally, the two MSE models were evaluated using the test set for sensitivity and specificity. Sensitivity and specificity were 85% and 84% for MSE & Adam, and 67% and 77% for MSE & SGD, respectively. Moreover, PABAK values for agreement with the correct label were 0.69 and 0.43 for MSE & Adam and MSE & SGD, respectively. Similar to the training and validation, MSE and Adam can recognize postures in the test set and demonstrate generalization performance on unknown data.

According to the results, the MSE & Adam model, in particular, can serve as a useful assessment tool for screening inspections.

## Figures and Tables

**Table 1:** Details of the main library versions used for the model development.

Name	Version
Python	3.6.10
Cudatoolkit	10.1
Cudnn	7.6.4
Kares	2.3.1
TensorFlow-gpu	2.1.0

**Table 2:** The cross-tabulation matrix of the correct and output labels across different datasets: (**a**) training set; (**b**) validation set; (**c**) test set.

(**a**)						
		Correct label				
		MSE & Adam			MSE & SGD	
		Ideal	Non-ideal		Ideal	Non-ideal
Output label	Ideal	2427	87		2177	717
	Non-ideal	57	2913		307	2283
(**b**)						
		Correct label				
		MSE & Adam			MSE & SGD	
		Ideal	Non-ideal		Ideal	Non-ideal
Output label	Ideal	735	156		665	339
	Non-ideal	93	844		163	661
(**c**)						
		Correct label				
		MSE & Adam			MSE & SGD	
		Ideal	Non-ideal		Ideal	Non-ideal
Output label	Ideal	696	149		636	331
	Non-ideal	132	851		192	669

**Table 3:** Performance metrics (accuracy, sensitivity, and specificity) of two models across different datasets: (**a**) training set; (**b**) validation set; (**c**) test set.

(**a**)			
	MSE & Adam (%)		MSE & SGD (%)
Accuracy	97		81
Sensitivity	97		76
Specificity	98		88
(**b**)			
	MSE & Adam (%)		MSE & SGD (%)
Accuracy	86		73
Sensitivity	84		66
Specificity	89		80
(**c**)			
	MSE & Adam (%)		MSE & SGD (%)
Accuracy	85		71
Sensitivity	85		67
Specificity	84		77

## Data Availability

The data are available from the corresponding author upon request.
